# Is artificial intelligence capable of generating hospital discharge summaries from inpatient records?

**DOI:** 10.1371/journal.pdig.0000158

**Published:** 2022-12-12

**Authors:** Kenichiro Ando, Takashi Okumura, Mamoru Komachi, Hiromasa Horiguchi, Yuji Matsumoto

**Affiliations:** 1 Graduate School of Systems Design, Tokyo Metropolitan University, Tokyo, Japan; 2 Center for Advanced Intelligence Project, RIKEN, Tokyo, Japan; 3 National Hospital Organization, Tokyo, Japan; 4 School of Regional Innovation and Social Design Engineering, Kitami Institute of Technology, Hokkaido, Japan; King’s College London, UNITED KINGDOM

## Abstract

Medical professionals have been burdened by clerical work, and artificial intelligence may efficiently support physicians by generating clinical summaries. However, whether hospital discharge summaries can be generated automatically from inpatient records stored in electronic health records remains unclear. Therefore, this study investigated the sources of information in discharge summaries. First, the discharge summaries were automatically split into fine-grained segments, such as those representing medical expressions, using a machine learning model from a previous study. Second, these segments in the discharge summaries that did not originate from inpatient records were filtered out. This was performed by calculating the n-gram overlap between inpatient records and discharge summaries. The final source origin decision was made manually. Finally, to reveal the specific sources (e.g., referral documents, prescriptions, and physician’s memory) from which the segments originated, they were manually classified by consulting medical professionals. For further and deeper analysis, this study designed and annotated clinical role labels that represent the subjectivity of the expressions and builds a machine learning model to assign them automatically. The analysis results revealed the following: First, 39% of the information in the discharge summary originated from external sources other than inpatient records. Second, patient’s past clinical records constituted 43%, and patient referral documents constituted 18% of the expressions derived from external sources. Third, 11% of the missing information was not derived from any documents. These are possibly derived from physicians’ memories or reasoning. According to these results, end-to-end summarization using machine learning is considered infeasible. Machine summarization with an assisted post-editing process is the best fit for this problem domain.

## Author summary

Medical practice necessitates significant paperwork; thus, the automated processing of clinical records can reduce the burden on medical professionals. To this end, some research efforts have attempted to achieve automatic summarization of inpatient records collected by physicians when they hospitalize patients. This study investigated whether discharge summaries can be constructed automatically from inpatient records to facilitate further processing. For this purpose, each piece of information in the discharge summaries is manually labeled to determine whether it originated from inpatient records. If not, possible sources were attempted to identify. The results revealed that 61% of the information in the discharge summary was derived from inpatient records, whereas the remaining 39% was derived from other sources. These external sources included patient’s past clinical records (43%) and patient referral documents (18%). Furthermore, 11% of the information did not originate from any document, indicating that it was possibly derived from physicians’ memories or reasoning. This suggests that a fully automated generation of discharge summaries is considered infeasible, and future research efforts must be directed toward semi-automated generation aimed at optimal human-machine collaboration in the authoring of discharge summaries.

## 1 Introduction

Artificial intelligence technology has been increasingly applied in various fields of medicine [1–7]. Its application in clinical texts is expected to improve the efficiency of paperwork [8–10], which has become a heavy burden for medical professionals. A recent study found that family physicians spent 5.9 h of their 11.4 h workday on electronic health records (EHRs) [11]. In 2019, 74% of physicians spent more than 10 h per week on paperwork and administration [12]. Another study reported that physicians spent 26.6% of their daily working time on documentation [13]. As physicians are busy with such clerical work, automatic generation of documents would relieve their burden. In this regard, hospital discharge summaries can be promising targets for automation because daily inpatient records are already available in these systems. Computers can efficiently support physicians by generating discharge summaries from inpatient records.

In natural language processing (NLP), various summarization techniques have demonstrated high accuracy in summarization benchmarks [14–19]. These technologies can be applied to summarizing inpatient records. Therefore, some studies on the automated generation of the whole discharge summary have been conducted [20–26]. Whether artificial intelligence can generate hospital discharge summaries from inpatient records remains an open question. To address this issue, it is important to find the source of the information expressed in the discharge summaries. If physicians rely on their memory, it would be difficult to automatically generate a discharge summary solely from inpatient records, even with a top-performing summarization technique.

**Fig 1:**
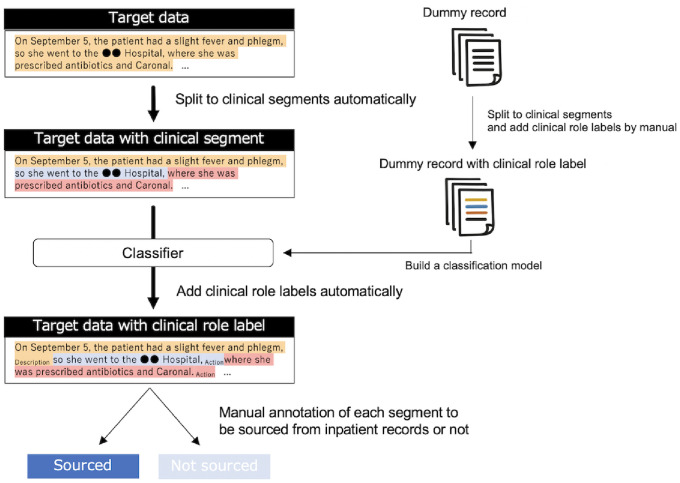
Proposed framework of our study. The colored blocks in the dummy record represent the clinical segment developed in previous study, where the sentence is split by medical sense [20].

Therefore, we designed the study to investigate the information sources of the discharge summaries (Fig 1). First, the discharge summaries are split into fine-grained segments such that each segment contains a single piece of medically meaningful information. Second, medical professionals manually classified each description from discharge summaries to determine whether it originated from daily inpatient records. Using manual classification, expressions that are completely different in appearance but semantically equivalent can be accurately identified. Finally, an in-depth analysis of the expressions in discharge summaries that could not be reconstructed from daily inpatient records was conducted. For this purpose, *clinical role labels* are defined, which indicate the type of medical subject to which a description refers. To overcome the problems of large and strictly privacy-sensitive target data containing raw patient information, a small dataset of dummy health records was annotated, and an automatic classification model was built.

The remainder of this paper is organized as follows. Section 2 presents related work. Section 3 describes the materials used. Section 4 discusses the manual annotation process and the classifier for clinical role labels. Here, multitasking was used to alleviate the problem of data sparseness and show that multiple granularity labels positively influence each other when used to train the model simultaneously. Section 5 presents the experiments and the results. Section 6 discusses the results of the study. Finally, Section 7 concludes this paper.

## 2 Related work

Automatic summarization is a well-studied topic in NLP [16–19, 27, 28] and has two main approaches: extractive and abstractive summarization. The former method extracts content from the source text and combines it to generate a summary. The text of the generated summary was wholly derived from the source and did not contain new content. The latter method generates a summary by creating new content based on the source using some algorithms. The algorithms used in previous studies include sentence compression [29], sentence fusion [30, 31], and sentence revision [32]. However, encoder-decoder architectures are commonly used at present [16–19]. In the medical field, extractive summarization methods are commonly used for knowledge acquisition of clinical features, such as diseases, prescriptions, and examinations.

In recent years, NLP in the medical domain has paid more attention to unstructured text than structured text. Tasks involving unstructured text are generally noisier and more difficult than those using structured text. Most previous NLP techniques for unstructured medical texts have focused on normalization and prediction, such as ICD (International Classification of Diseases) codes, mortality rates, and readmission risks [33–39]. They target extracting or predicting important information from the input rather than the intended summarization, such as outputting a document. In addition, some studies have attempted to retrieve important information from EHRs [40–43], such as diseases, examination results, and medications, while collecting fragmented information and not attempting to generate contextualized passages. Other studies generated several key sentences from EHRs to provide physicians a quick grasp of the main points [44–47]. Most previous studies that generated a whole discharge summary used structured data as input [48–50]. Some recent studies have attempted to generate a complete discharge summary from the input of free-form inpatient records similar to ours [21–26]. While some studies employed extractive methods [23–26], in other studies, the encoder-decoder architecture of the neural model was used to generate sentences for abstractive summarization [21, 22], with a limited number of studies.

Because abstractive summarization can generate more flexible summaries, it has become a major approach in automatic summarization research [14–19]. However, abstractive summarization may sometimes unintentionally generate unfaithful descriptions known as **hallucinations**. Summaries with hallucinations are fluent [51], but hallucinations degrade the summary quality. Therefore, they have attracted attention in the field [52–57]. Maynez et al. (2020) classified hallucinations into two types: intrinsic and extrinsic [51]. Intrinsic hallucination is a phenomenon in which the concept or term itself is in the source documents; its synthesis misrepresents the information in the source, and the meaning becomes inconsistent. Extrinsic hallucination is content that is neither supported nor contradicted by the source and is caused by source documents with poor information. Therefore, the analyses of extrinsic hallucinations in previous studies are almost equal to our investigation of the information sources in discharge summaries. In discharge summaries, complementary statements that are not explicitly stated in inpatient records may be inserted; however, they can be inferred easily by medical professionals. This study does not consider this to be a hallucination if the information can be inferred, although previous studies have defined hallucinations more rigorously. To the best of our knowledge, this study is the first attempt to address hallucination problems in the summarization of clinical narratives.

## 3 Health record datasets

The target data were the National Hospital Organization’s Clinical Data Archives (NCDA) [58], operated by the National Hospital Organization (NHO), Japan. The archive is the largest collection of multi-organizational health records in Japan and stores replicated EHR data from 66 national hospitals. It has become a valuable data source for multi-institutional research in the country. Our dataset is a randomized subset of archives, including 24,641 cases collected from five hospitals that belong to the NHO. Hereinafter, this study refers to this dataset as **NHO data**. Each case included inpatient records and discharge summaries of a patient in the internal medicine department. The inpatient records in this study refer to the main contents of the EHRs that describe patients’ progress during hospitalization. This is similar to the document called “progress note” in MIMIC-III, which is a large-scale medical dataset in English [59]. In this study, only the free text area was used to summarize the clinical text automatically. This study did not aim to collect meta-information, such as basic patient information, which can be compiled automatically.

To create a classifier of clinical role labels, this study used another dataset, a **dummy record**. This was a paired dataset of 108 inpatient records and discharge summaries created for research purposes without privacy restrictions. A dummy record was used to develop the annotation guidelines for clinical role labels (explained below) and to build a classification model that was used to label the target data.

For preprocessing, each sentence in the dataset was first split by end marks and line breaks. A primitive approach was adopted because the complex sentence-splitting model might introduce biases in subsequent analyses, as the clinical documents used in this study were noisy. Each sentence is then split into *clinical segments* representing the minimal units of medical concepts to capture fine-grained information [20]. In this process, the model built by Ando et al. (2022) was used for the automatic assignment of clinical segments. This is another machine learning model trained in another study [20].

This study was conducted under the IRB approval of the Institute of Physical and Chemical Research (RIKEN) Japan (IRB Approval No. Wako3 2019-22), which has been collaborating with the National Hospital Organization. NCDA ensures patient privacy and informed consent in the following manner. First, the EHR data policy is posted at national hospitals in the form of notices. Second, patients who disagree with the policy are expected to notify the hospital in a written manner using an opt-out form to be excluded from the archive. Similarly, minors and their parents can turn in the opt-out form at will. Third, researchers must submit research proposals to an institutional review board before conducting a study on archives. Once the study is approved, data are extracted from the NCDA and anonymized to construct a dataset for a particular study. The data are accessible only in a secure room at the NHO headquarters, and only statistics can be obtained from the secured room to protect patients’ privacy.

## 4 Automatic clinical role labelling

For the analysis of the main experiment, the expressions in the NHO data must be examined in depth. However, checking expressions for cost and privacy manually is unrealistic. Therefore, expression labels under physician supervision were defined, and a classification model for labeling automation was developed in this study.

### 4.1 Clinical role labels

To analyze the summarization patterns, expressions that appeared in clinical documents were checked, and their types were defined. In this definition, it is assumed that the clinical facts are interpreted by physicians, and the processing progresses in this order in the summarization of inpatient records. For example, physicians may perform physical and laboratory examinations during the early stages of hospitalization. They recorded the results in the inpatient records as facts. Subsequently, evaluations of the test results, diagnoses, treatment plans, etc. would be performed by physicians based on their interpretations. Therefore, there must be a gradation in subjectivity in the descriptions that appear in inpatient records and summaries. Subjective descriptions may include interpretations of objective information in the source record. Based on this assumption, the clinical role labels are defined (Table 1). All definitions were designed under the supervision of a physician.

**Table 1:** Details of the clinical role label. It shows label names, brief explanations, and examples in discharge summaries.

Label	Explanation	Example
**Low Subjectivity Labels**		
*Description*	Past events and status.	Only pneumococcal urine antigen test results are positive.
*Action*	Past actions.	Discharged.
*Others*	Meaningless segments.	However, “
**Middle Subjectivity Labels**		
*Result*	Comments as seen, but can change slightly from person to person.	Infiltration shadow in the lower right lung field
*Undefinable*	Unclear whether descriptions are future plans caused by Japanese linguistic characteristics.	4月 10 日に入院。 〈Hospitalized or will be hospitalized on April 10.〉
**High Subjectivity Labels**		
*Evaluation*	Reasoning from facts.	Because it was considered an acute exacerbation of interstitial pneumonia
*Diag*	Clinical or definitive diagnosis.	Clinically diagnosed with small-cell lung cancer
*Plan*	Future treatment plans.	The patient was scheduled for long-term PCI.
*Nonfact*	Hearsay and assumptions, etc.	Considering his advanced age and limited life expectancy
*Probable*	Probabilistic expressions.	Suspected renal abscess or renal cell carcinoma.

#### 4.1.1 Low subjectivity labels

First, *low subjectivity labels* are defined to include *Description*, *Action*, and *Others* labels. They consisted of objective facts and formed the basis of clinical records and discharge summaries.

***Description*** labels comprise the content of past events and statuses. These are the fundamental contents of clinical records. For example, observations of patients, physical findings, test results, and paraphrasing of test results (e.g., “high blood pressure” instead of “Blood Pressure:180/90”), and past episodes fell under this category. The paraphrases included in this label are limited to the conversion of expressions without any interpretative comments. The *history of present illness* section mostly consists of the *description* label because it is based on patients’ past episodes.

The ***Action*** label comprises the contents of someone’s past actions (e.g., “hospitalized,” “prescribed,” and “discharged”). These were mostly medical treatment records. Here, action verbs can be active, passive, or other voices, and any form is acceptable for action content.

The ***Others*** label comprises meaningless content from a medical perspective. Typical examples are dates, item names, parentheses before and after a segment, etc. (e.g., “【現病歴】” 〈“[history of present illness]”〉). Although these symbols are objective descriptions, they do not contain information about patients. Thus, this study reserved a class for such cases to simplify further processing.

#### 4.1.2 Middle subjectivity labels

Second, *middle subjectivity labels* are defined, which include *result* and *undefinable* labels. In clinical documents, determining the subjectivity of some descriptions is difficult; these categories are devised to accommodate such cases and maintain the quality of annotations for high and low labels.

The ***Results*** label comprises content that is slightly subjective to healthcare providers. For example, physicians record abnormalities and interpret images in radiological reports. However, they often comprise qualitative descriptions and objective expressions, which results in a combination of subjectivity and objectivity. Other examples include changes in test values (e.g., “improvement” and “worsening”) that can also be influenced by physicians’ subjectivity. Clinical documents may contain expressions that are difficult to categorize as factual or subjective. This label was intended as a buffer to cover borderline cases.

The ***Undefinable*** label comprises content that is unclear whether it is a reference for a future plan. In Japanese, to write a concise sentence, predicates are often transformed into nouns (e.g., “退院した” 〈“Discharged.”〉 → “退院” 〈“Discharge.”〉 “検査する” 〈“to examine.”〉 → “検査” 〈“examination.”〉). In such cases, whether the examples refer to past or future plans remains unclear.

#### 4.1.3 High subjectivity labels

Third, *high subjectivity labels* are defined, including *evaluation*, *diag*, *plan*, *nonfact*, and *probable* labels. This class comprises information that is a hypothetical or subjective statement by the writer. Such content is produced by accepting clinical findings as inputs, and then inferences, external knowledge, and personal insights are used to generate the outputs. This is the primary content of the *clinical course section*, which appears to be the most difficult part to summarize automatically.

The ***Evaluation*** label comprises content that is discussed and reasoned about, findings, test results, and events. This category is another core element of clinical records. A general example is the list of test results followed by discussion of the findings. In clinical texts, a description of *evaluation* may accompany a trailing *diag* description and can be inseparable if the descriptions are abbreviated (e.g., “diagnosed as COVID-19 based on the severe clinical course”). As our labeling framework allows multiple labels, a sentence may contain both *evaluation* and *diag* labels. However, this case rarely appeared in our annotation; thus, the *diag* label was prioritized over the *evaluation* label.

***Diag*** labels were used for the clinical diagnosis. Although a definitive diagnosis can be performed objectively, the diagnosis relies on objective findings. Therefore, in this classification, diagnostic descriptions were considered low-objectivity classes. This label is similar to the *evaluation* label; it is also a core element of the clinical record. Note that there are medical concepts that can be both symptoms and diseases, depending on the context, such as “dyspnea.” Such borderline cases are assigned to the *result* label to avoid contamination of the *diag* labels.

The ***Plan*** label was assigned to expressions that explicitly refer to future plans. In Japanese, such expressions often comprise certain terms, such as “予定(schedule)” and “計画(plan).” These are mainly written at the bottom of the inpatient records. They sometimes refer to the next scheduled visit and referral source that the patient will visit after discharge.

The ***Nonfact*** label comprises content written with hearsay or assumptions; however, it does not belong to any other label. Some characteristic words in Japanese, such as “if,” “consider,” and “say that,” indicate that the content is not based on fact.

Finally, the ***probable*** label comprises clearly subjective content, such as “doubt” or “possibility.” This label must have a multilabel structure as it can be added to any content In this case, all contents labeled as *probable* are classified as *high* in the subjectivity label, regardless of the original label, because their information becomes subjective.

#### 4.1.4 Annotation

Dummy records were annotated with clinical role labels. Initially, the sentences were split and were decomposed into 3,761 clinical segments, as described in Section 3. The annotation was conducted by two clinical workers, and the agreement rate was calculated as the accuracy, which was 0.790. The distribution of clinical role labels is shown in the left half of Table 2.

**Table 2:** Distribution of the clinical role and subjectivity labels. The labels in the dummy record were manually annotated by clinical workers and the NHO data were automatically annotated.

Subjectivity	Clinical role	Dummy record		NHO data	
		Number of segments (%)		Number of segments (%)	
Low	Description	1,463 (37%)	2,324 (61%)	484,385 (32%)	917,724 (60%)
	Action	797 (20%)		183,245 (12%)	
	Others	65 (2%)		241,729 (16%)	
Middle	Result	306 (8%)	646 (17%)	160,118 (11%)	401,049 (26%)
	Undefinable	340 (9%)		258,939 (17%)	
High	Evaluation	278 (7%)	844 (22%)	45,043 (3%)	205,671 (14%)
	Diag	255 (6%)		60,772 (4%)	
	Plan	264 (7%)		53,389 (4%)	
	Nonfact	82 (2%)		12,511 (1%)	
	Probable	133 (3%)		24,313 (2%)	

The most common label was *description*, followed by *action*. Both are past facts that appear to be appropriate considering the original purpose of the medical records, which was to record the medical treatment process. In addition, *description* was twice as common as *action*, suggesting that recording the past status plays a major role in clinical records. For high subjectivity, *evaluation*, *diag*, and *plan* were nearly the same in number, whereas *nonfact* and *probable* were relatively low. This suggests that the medical records consist of an equal amount of evaluation of findings and test results, clinical diagnosis, and plans for future treatment.

### 4.2 Automation of labeling

Using the annotated dummy records, this study trained a classification model that was used to classify the NHO data shown on the right-hand side of Table 2.

#### 4.2.1 Classification model

**Fig 2:**
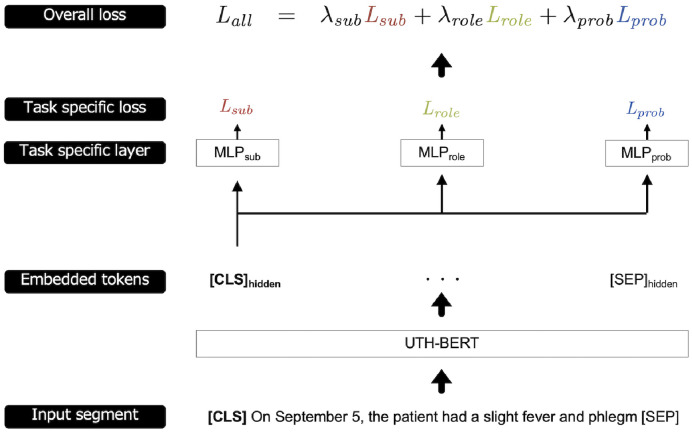
Overview of the classification model for subjectivity, clinical role, and probable label. Each of the three labels is defined as three tasks. Input segments are fed to UTH-BERT, and then the outputs to the specific layers. Finally, the loss scores of three tasks are calculated and combined to obtain the overall loss score.

An overview of the proposed model is shown in Fig 2. As the basic architecture for classification, this study adopted a pretrained neural model, BERT (Bidirectional Encoder Representations from Transformers) [60]. Because its parameters are learned from a large number of documents in advance, BERT is known to achieve good accuracy even with few training samples. In this study, UTH-BERT was used [61], an improved version of BERT that was pre-trained on clinical records from the University of Tokyo Hospital. In contrast to previous Japanese BERT models [62–64], which were pre-trained mainly on web data such as Wikipedia, UTH-BERT was expected to perform better on documents in our target domain. (For more detailed architecture, training methods, and performance of UTH-BERT, see previous papers [60, 61, 65].)

This study also adopted a multitask learning framework. Multitask learning achieves improved performance by exploiting the relationship between labels and is considered to provide various benefits (e.g., regularization, eavesdropping, and data augmentation [66]). In our study, three labels (i.e., subjectivity, clinical roles, and probable labels) were assigned to a clinical segment, and multitask learning compensated for the small data volume of the dummy records by virtually multiplying the labels used for learning. Subjectivity prediction can also aid in a more complex clinical role prediction task.

The processing pipeline operates as follows: a clinical segment split from the target dataset is input into the BERT. The input segment was previously tokenized by WordPiece [67] and provided with tokens “[CLS]” for the head and “[SEP]” for the tail. Then, the [CLS]_hidden_ vector from the final layer of BERT is obtained and inputted to a separate three-layer perceptron for each of the three labels. It calculates the cross-entropy loss value based on the gold and predicted labels and obtains three loss values. The model was trained using the weighted sum of the three loss values as the overall loss. In this process, BERT is trained only on the parameters of the final layer. The weighted sum *L*_*all*_ is formulated as

$$\begin{array}{ccc} L_{\text{all}} & = & \lambda_{\text{sub}}L_{\text{sub}}+\lambda_{\text{role}}L_{\text{role}}+\lambda_{\text{prob}}L_{\text{prob}}, \end{array}$$ (1)

where *L*_*sub*_, *L*_*role*_, and *L*_*prob*_ are the loss values for subjectivity, clinical role, and probable, respectively, and λ_*sub*_, λ_*role*_, and λ_*prob*_ are the hyperparameters of the corresponding weights. λ_*sub*_, λ_*role*_, and λ_*prob*_ were normalized and summed to 1.

In the implementation, this study employed UTH-BERT, which was pre-trained using the method of whole-word masking. In addition, the Adam optimizer was used [68] for 20 epochs, and the learning rate *η* = 0.00001. Based on the available memory and training performance, the batch size is set to 32. The setup of the other hyperparameters was the same as that in UTH-BERT. For the development and testing data, 300 samples were randomly selected from the dummy records, and the remaining examples were used as training data. The average results of three training runs with different seeds are reported.

#### 4.2.2 Results of classification

The hyperparameter search and classification results are presented in Tables 3 and 4, respectively. The model performance was evaluated using the F1 score against the correct labels. The F1 score is the harmonic mean of recall and precision. Recall is formulated as $\frac{\text{TP}}{\text{TP}+\text{FN}}$, where TP is the number of true positives and FN is the number of false negatives. In addition, precision was formulated as $\frac{\text{TP}}{\text{TP}+\text{FP}}$, where FP is the number of false positives. Let recall be R and precision be P, $\text{F}1=\frac{2RP}{R+P}$. In the hyperparameter search, each weight was changed by 0.25 and grid-searched to find the optimal value. The F1 scores with individual labels are found in the columns in which λ_*sub*_, λ_*role*_, and λ_*prob*_ are 1. This study found that multiple-label settings were always better than single-label settings.

**Table 3:** Results of a hyperparameter search. Three labeling tasks are conducted as independent tasks, and the weights of the tasks are slid by 0.25 to find the optimal value in multitask learning. Experiments are conducted using dummy records.

Hyperparameters																
Subjectivity																
λ_*sub*_	1	0.75		0.5			0.33	0.25				0				
Clinical role																
λ_*role*_	0	0.25	0	0.5	0.25	0	0.33	0.75	0.5	0.25	0	1	0.75	0.5	0.25	0
Probable																
λ_*prob*_	0	0	0.25	0	0.25	0.5	0.33	0	0.25	0.5	0.75	0	0.25	0.5	0.75	1
Accuracy																
F1_sub_	0.830	0.834	0.825	0.846	**0.862**	0.856	0.856	0.859	0.844	0.840	0.823	0.574	0.534	0.854	0.406	0.598
F1_role_	0.060	0.751	0.069	0.765	0.767	0.050	0.771	**0.774**	0.745	0.765	0.053	0.747	0.722	0.772	0.737	0.008
F1_prob_	0.382	0.397	0.951	0.967	0.965	0.962	**0.969**	0.425	0.967	**0.969**	0.967	0.395	0.960	0.384	**0.969**	0.962

**Table 4:** Results of automatic labeling using dummy record. The hyperparameters of λ_*sub*_, λ_*role*_, and λ_*prob*_ are 0.5, 0.25, and 0.25 for subjectivity; 0.25, 0.75, and 0 for clinical role; and 0.33, 0.33, and 0.33 for probable label.

**Roles**	**Precision**	**Recall**	**F1**
Description	0.86	0.87	0.87
Action	0.88	0.81	0.84
Others	1.00	0.43	0.60
Result	0.65	0.65	0.65
Undefinable	0.55	0.77	0.64
Evaluation	0.61	0.83	0.70
Diag	0.79	0.88	0.83
Plan	0.81	0.65	0.72
Nonfact	0.00	0.00	0.00

**Probable**	**Precision**	**Recall**	**F1**
Positive	0.60	0.55	0.57
Negative	0.98	0.99	0.98

**Subjectivity**	**Precision**	**Recall**	**F1**
Low	0.92	0.90	0.91
Middle	0.66	0.73	0.70
High	0.85	0.84	0.84

In the detailed classification results for each label, this study found that the model could be classified with much higher accuracy for high and low subjectivities. The classification accuracy is lower for middle subjectivity, which is not surprising because this label includes ambiguous segments that improve annotation quality. This study did not use the middle label for further analysis. Furthermore, in the detailed labels for high and low subjectivities, this paper found that *others* and *nonfact* are low. This was because of the small sample size of these labels. For the same reason, the *probable* label is less accurate.

The distribution of automatically assigned labels is shown in the right-hand half of Table 2, along with the distribution of the dummy records. This study found that low subjectivity was present in the same proportion as in the dummy record. Upon comparing middle and high subjectivity, the statistics show that middle subjectivity is more common. This is because many formatting expressions exist in NHO data, such as examination results, dates, and times. Overall, the distributions of the dummy records and NHO data were mostly consistent. This suggests the appropriateness of the automated labeling process.

## 5 Main experiments and analysis

### 5.1 Classification of unsourced segments

**Fig 3:**
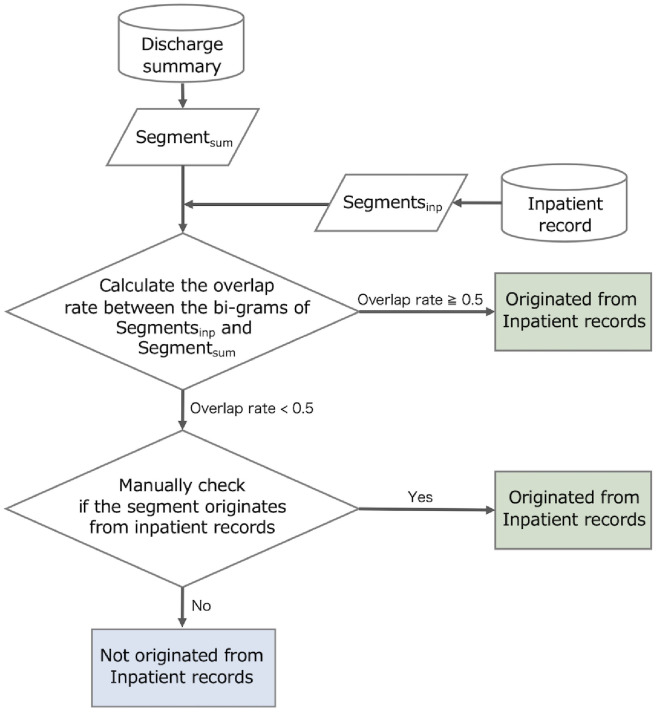
Our annotation flowchart of the source origin. The source origin is manually determined in two steps using pre-filtering.

A two-step approach was employed to measure the proportion of segments in the discharge summaries originating from inpatient records. A flowchart of the proposed process is shown in Fig 3. First, segments in the discharge summaries were automatically classified using a simple matching algorithm for inpatient records. If the exact segments were found in the records, they were obtained from them. However, the naive algorithm cannot handle synonymous expressions, thus preventing a fully automated classification. Therefore, in the second step, this study employed the manual annotation of segments considered unsourced by automated classification. The target data comprised 772 segments extracted from 24 randomly selected documents. These documents were selected from the five hospitals in the NHO. Symbols from the system output, dates, and other symbols were excluded from this task because they were meaningless in the annotation.

**Fig 4:**
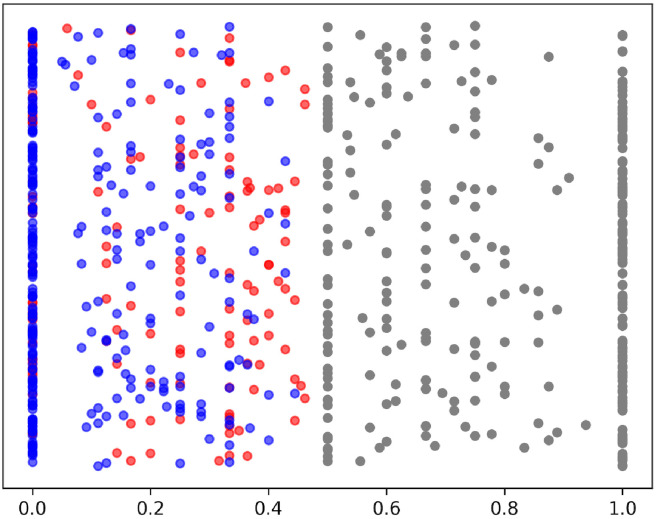
Origin rate of segments in discharge summaries against the inpatient records. Distribution of origin rates using bi-grams from the randomly sampled data. Red, blue, and gray dots are sourced, unsourced, and filtered out segments, respectively. Note that symbols and segments categorized as middle subjectivity are excluded. The y-axis values were randomly generated from a uniform distribution of visibility.

In the first step, word-based bi-grams were used to determine whether the segments in the summaries were sourced from inpatient records. To this end, a bi-gram set was created from all inpatient records, and a list of bi-grams from each discharge segment summary was created. Subsequently, coverage with the bi-gram set from inpatient records was measured. The bi-gram method was adopted because the distribution of the coverage ratio was closer to uniform across the entire value range (Fig 4). For simplicity, the classification threshold was set to 0.5, which was validated through analysis.

In the second step, segments with coverage ratios of less than 0.5 were manually annotated. A total of 408 segments were used for annotation. The task involved comparing each segment against inpatient records and labeling whether information in the segment was provided in the source. This task required both medical and clinical knowledge. Annotations were performed by an expert in NLP (Author K.A) and two medical professionals. To relieve the burden on annotators, the author first assigned temporary labels to all the data. Subsequently, a domain expert checked the labels and corrected them if they appeared wrong. Finally, another expert checked and fixed the labels. The inter-annotator agreement rate was 0.952, indicating the validity of the labels.

**Fig 5:**
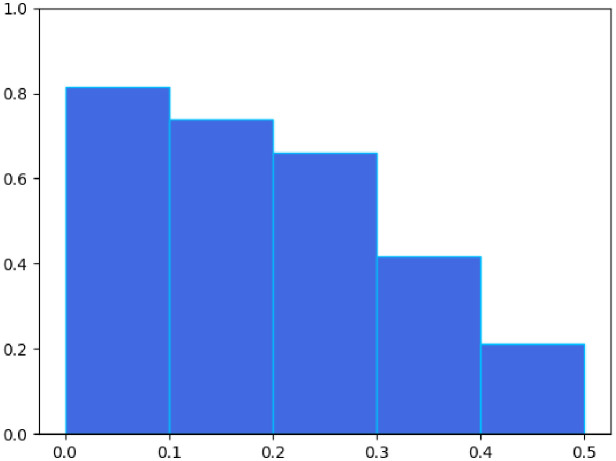
Origin rate of segments in discharge summaries against the inpatient records. Proportion of unsourced segments appearing in manually annotated data. The y-axis is the value averaged every 0.1 steps for segments with origin rates less than 0.5, as shown in Fig 4.

The automatic and manual classification results for the annotations are shown in Figs 4 and 5, respectively. The bi-gram match rate was divided into five intervals from 0 to 0.5 to confirm the validity of our threshold. The probability of the presence of unsourced segments in these intervals was measured (Fig 5). This probability decreases as it approaches 0.5, with a low probability near 0.5, equal to 0.2. This indicates that our threshold of 0.5 is sufficient to cover the segments suspected to be unsourced.

This study checked the amount of information in hospital discharge summaries that could not be reproduced from the inpatient records. Table 5 shows the detailed label results. The overall percentage of sourced segments was 61.3%, indicating that 38.7% of the information in the discharge summary was obtained from external documents other than inpatient records. In addition, the document-based unsourced rate, including at least one unsourced segment in a document, amounts to 87%. Considering the unsourced rate for each subjectivity, this study found that the unsourced probability is higher for high subjectivity than for low subjectivity. This suggests that statements involving subjectivity do not rely much on documents and are written by physicians themselves. For clinical role labels, *diag* and *probable* were relatively high. This indicates that the core of medical practice, such as diagnosis and prediction based on facts, is often generated in discharge summaries.

**Table 5:** Rate of unsourced segments in detailed labels. Because the clinical role and the subjectivity labels are automatically added as different tasks, the subjectivity label is not a weighted average of the clinical role labels. In contrast, “All” is a weighted average of low and high subjectivity.

Subjectivity	Clinical Role	Unsourced rate			
Low	Description	0.369	0.376	All	0.387
	Action	0.403			
	Others	0.304			
High	Evaluation	0.487	0.439		
	Diag	0.529			
	Plan	0.422			
	Nonfact	0.429			
	Probable	0.583			

Discharge summaries typically comprise descriptions of pre-hospital episodes and in-hospital information. The “pre-hospital” part consists of past medical history, a history of present illness, and results of examinations at the time of admission, whereas the “in-hospital” part comprises all patient descriptions obtained after admission. Table 6 summarizes the unsourced and high subjectivity rates in the pre-hospital and in-hospital settings. The unsourced rates for the “pre-hospital” and “in-hospital” parts are 0.434 and 0.318, respectively, illustrating the higher rate in the “pre-hospital” part. This is plausible because the hospitals that participated in this survey were central hospitals, and most patients visited them by referral. These hospitals had referral letters and past clinical records that could be used for summarizing inpatient records (more details are provided in Section 5.2). Additionally, the pre-hospital section had a lower percentage of high subjectivity segments. This reflects that the content of this section is mainly patient history. In contrast, the in-hospital section had a higher percentage of high-subjectivity segments, reflecting content such as speculation, planning, and diagnosis, which generally occur during hospitalization.

**Table 6:** Rate of unsourced and high subjectivity segments in two sections. The sections “Pre-hospital” and “In-hospital” include descriptions of patients before and after admission.

	Unsourced rate	High subjectivity rate
Pre-hospital	0.434	0.130
In-hospital	0.318	0.235

### 5.2 Origin of unsourced information

The results suggest that physicians refer to various documents and inpatient records when preparing discharge summaries. This section identifies the sources of information that appear in the discharge summaries in addition to the inpatient records. To this end, 14 labels were developed to classify sources of information: *patient referral documents, outpatient clinical records, emergency room records* and *patient’s past clinical records* (which cannot be categorized in other labels of past records and mainly include patient’s past inpatient records) are descriptions of past history. *Prescriptions, nursing records, examination results, ECG reports, rehabilitation reports, surgical operation notes* and *anesthesia records* are descriptions of the current admission. *Other patients’ clinical records, other documents*, and *information not derived from any documents* (i.e., a physician’s memory or inference) are the descriptions of the others.

For example, drug information written in quantitative form was labeled as *prescriptions*. Events during rehabilitation were labeled as *rehabilitation reports*. The admission episodes of patients from the emergency department were labeled as *emergency room records*. Doctors’ impressions and inferences are labeled *not derived from any documents*. These labels may appear lengthy; however, they facilitate further insight into the origin of the information written in the discharge summaries. Expressions labeled *not derived from any documents* included information that could not be recorded during the hospital stay, such as descriptions of the times of discharge and post-discharge schedules. They also included physicians’ perspectives on diagnostic approaches and treatment options. They may also contain excessive abbreviations for the hospital stay such as “no significant change,” descriptions of normal conditions such as “able to eat,” and omission of details of standardized protocols such as “fluids and antibiotics.” Annotation was performed by including two medical professionals, as described in Section 5.1. The inter-annotator agreement rate is 0.938. Such a high score indicates the objectivity of the designed annotation labels with a physician.

The statistical results are listed in Table 7. Overall, this study found that 43.3% of the new information was derived from the patient’s past clinical records. When patient referral documents are included, the coverage of the new information is 61.7%, which suggests that the availability of these two types of documents can complement 61.7% of the missing information.

**Table 7:** List of source documents that the annotators selected for each piece of information labeled as *not sourced from inpatient records*. The numbers indicate the percentage of external documents in each section.*Low subj*, *High subj*, *Pre-hosp*, and *In-hosp* in the table show the distribution of assumed external sources for segments classified as unsourced. Because it has a multi-label structure, each segment may have multiple source labels, and the percentile is calculated against the total number of assigned labels.

	Documents	All (%)	Low subj (%)	High subj (%)	Pre-hosp (%)	In-hosp (%)
Past history	Patient referral documents	18.4	19.5	14.5	21.4	12.3
	Outpatient clinical records	6.6	5.8	9.1	9.1	0.8
	Emergency room records	3.9	4.5	2.7	5.5	1.1
	Patient’s past clinical records	43.3	43.6	42.7	56.0	16.1
Current admission	Prescriptions	2.0	2.2	0.9	0.0	6.3
	Nursing records	1.5	1.8	0.0	0.0	4.5
	Examination results	5.7	5.8	6.4	0.5	17.6
	ECG reports	0.0	0.0	0.0	0.0	0.0
	Rehabilitation reports	1.5	1.8	0.0	0.0	4.5
	Surgical operation notes	0.7	0.4	1.8	0.0	2.3
	Anesthesia records	0.0	0.0	0.0	0.0	0.0
Others	Other patients’ clinical records	3.5	4.2	0.0	5.0	0.0
	Other documents	2.0	1.6	3.6	0.8	4.5
	Not derived from any documents	10.9	8.8	18.2	1.7	29.9

As a general trend, there were no significant differences between the two groups in the low and high subjectivity columns. However, in the *not derived from any documents* row, high subjectivity segments indicate a higher proportion (18.2%) than low subjectivity segments (8.8%). This indicates that, when physicians write summaries, they often add information based on reasoning rather than memory.

A characteristic difference was observed in the prehospital and in-hospital periods. The top four documents in the prehospital section describe the history of patient admission. This is the natural result of this function. Among these, the patient’s past clinical records showed a significantly high rate. This indicates that, in the hospitals studied in this paper, a large number of admitted patients were former patients rather than referrals. However, information not derived from any document was the most common item in the in-hospital section. This is also a natural function of the section because it is a place to fill in doctors’ perspectives.

### 5.3 Interpretation and generalizability of the results

**Fig 6:**
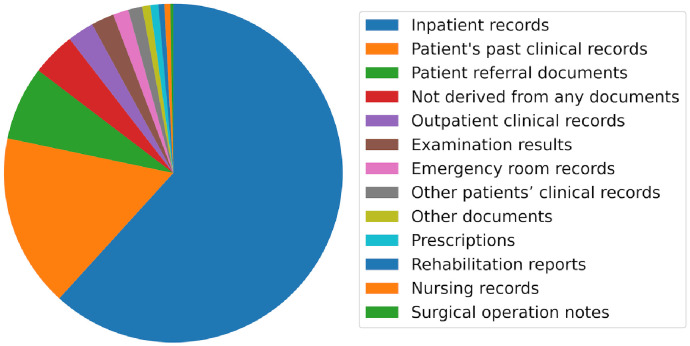
Breakdown of the information source in discharge summaries.

The analysis indicates a breakdown of the origin of information that appears in the discharge summaries (Fig 6). Information derived from inpatient records constituted 61% of the discharge summaries. The next most common source was the patient’s past clinical records (17%), and the third most common source was patient referral (7%) of the documents. To this point, 85% of the information in discharge summaries originates from documents associated with the patient. The fourth most common source was *not derived from any documents*, which explained 4% of the information sources.

As illustrated, physicians can refer to various documents, in addition to inpatient records, when they write discharge summaries. In this analysis, the number of target documents was limited because manual annotation was performed for accuracy. Although the analysis reveals that a substantial proportion of the contents in discharge summaries originate from sources other than inpatient records, the generalizability of the results should be verified. For this purpose, the variance between hospitals was analyzed and is listed in Table 8.

**Table 8:** Rate of unsourced and high subjectivity segments in institutions. Roman numerals indicate the five surveyed hospitals.

	Hospital				
	I	II	III	IV	V
Unsourced rate	0.596	0.289	0.231	0.461	0.360
High subjectivity rate	0.112	0.084	0.259	0.203	0.292

Focusing on the differences between hospitals, this study found that the unsourced rates differ greatly across hospitals. This difference can be ascribed to design differences in the documentation of electronic health record system vendors. These results suggest that hospital-specific bias must be considered when analyzing clinical narratives. Source availability may affect the way physicians write discharge summaries. Furthermore, the variation in the high subjectivity rates was limited, suggesting that the clinical reasoning process by physicians follows similar patterns across different types of facilities. In either case, the limited amount of data is a limitation of this study, and extending the studies to various types of institutions, probably with automated classification, would be valuable.

## 6 Discussion

This study investigated the origin of the information that appears in discharge summaries to evaluate the possibility of an automated summarization of inpatient records. The analysis results indicate that only 61% of the total information is derived from inpatient records, and 39% of the information originates from sources other than records. Manual evaluation by medical professionals identified past medical documents as the most common source of external information, such as patient referral documents and patient’s past clinical records. These two types of source documents accounted for 62% of the missing information. This study also found that 11% of the information contained speculation and post-discharge plans that were not derived from documents.

Previous studies indicate that automated summarization using a trained model from inputs with incomplete information for the target summary leads to hallucinations [53]. A previous study on news summarization using a dataset with an unsourced rate of 73% in document-based counts yielded a high incidence of extrinsic hallucinations [51]. In news summarization, the content is created from the source and supplemented by other news articles or common sense, which explains extrinsic hallucinations [53]. Our study revealed that the unsourced rate of expressions in the discharge summaries was 38.4% for the segment-oriented count and 87% for the document-based count. Therefore, if the dataset containing only inpatient records is used in the summarization of inpatient records, a higher incidence of hallucinations would be caused by the high unsourced rate. Considering the nature of healthcare, this result is unacceptable.

Clinical document summarization is inherently a multidocument summarization. Approximately 62% of the missing information could be generated if the patient’s past clinical records (43.3%) and patient referral documents (18.4%) were available. However, 11% of the information depends on the physician’s memory and clinical reasoning, and this portion is difficult to generate automatically. Therefore, automatic high-quality summarization using machine learning is considered infeasible, and machine summarization with a human post-editing process is the best solution for this problem.

A limitation of the present analysis lies in the volume of the target documents manually annotated and in the representativeness of the sampled target. A more thorough and detailed analysis might result in different statistics, and language differences must also be considered when applying the results to other languages. However, differences that may emerge in the additional analysis would be minor compared to the technical contributions of the present study. Extending the source material beyond inpatient records is necessary for the automated generation of discharge summaries. It is also necessary to improve the accuracy of abstractive summarization and present a draft that effectively elicits physicians’ reasoning and memory.

## 7 Conclusion

This study investigated whether artificial intelligence and natural language processing can automatically generate discharge summaries. The results indicate that the majority of the discharge summaries originated from sources other than patient records. The patients’ past clinical records and patient referral documents were the most and second-most external sources, respectively. This study found that a certain amount of external information was generated by the physician’s memory and clinical reasoning. The analysis suggests that the automated generation of discharge summaries is impossible using a naive collection of inpatient records. The generation of discharge summaries involves multiple document summarizations and clinical reasoning with undocumented information by physicians in charge of hospitalized care.

Undoubtedly, the automatic generation of discharge summaries could reduce the heavy burden on medical practice; thus, development in this field is highly desirable. Our results suggest that research efforts must be made to establish an optimal interaction between humans and machines for the efficient authoring of discharge summaries by incorporating generated drafts and post-editing assistance.
